# Solvent-free non-isocyanate polyurethane thermoplastic films from lignin and castor oil: toward sustainable packaging applications

**DOI:** 10.1039/d6ra02212j

**Published:** 2026-09-11

**Authors:** Kavya Ganesan, Srikanth Pilla, Kevin L. Simmons, James Sternberg

**Affiliations:** a Department of Food, Nutrition and Packaging Science, Clemson University Clemson South Carolina 29634 USA sternbe@g.clemson.edu; b Center for Composite Materials, University of Delaware Newark Delaware 19716 USA; c Department of Mechanical Engineering, University of Delaware Newark Delaware 19716 USA; d Department of Chemical and Biomolecular Engineering, University of Delaware Newark Delaware 19716 USA; e Department of Materials Science and Engineering, University of Delaware Newark Delaware 19716 USA; f Pacific Northwest National Laboratory Richland WA 99354 USA

## Abstract

Polyurethane (PU) films widely used in flexible packaging rely on petroleum-derived polyols and toxic isocyanates, raising significant environmental and safety concerns. Non-isocyanate polyurethanes (NIPUs), synthesized through the polyaddition of cyclic carbonates and amines, represent a promising alternative for developing safer and more sustainable polymer systems. In this study, fully bio-based thermoplastic NIPU films were prepared under solvent-free conditions using sebacic bis-cyclocarbonate (Seb CC), derived from castor oil, and lignin as a renewable aromatic component. Cyclocarbonated lignin (CC lignin) and native lignin were incorporated at varying Seb CC : lignin ratios (1 : 0.25–1 : 1) to evaluate the influence of lignin chemistry and loading on film structure and performance. The resulting films were characterized using FTIR, solid-state ^13^C CP/MAS NMR, thermal analysis (DSC and TGA), mechanical testing, permeability measurements (WVTR and OTR), scanning electron microscopy, and micro-computed tomography to investigate structure–property relationships. Lignin incorporation increased glass transition temperature, tensile modulus, and thermal stability, while significantly improving barrier performance. Native lignin formulations exhibited the lowest WVTR (0.33 g (100 in^2^·day)^−1^) and OTR (3.27–6.62 cc (100 in^2^·day)^−1^), approaching the performance of high-barrier biodegradable polymers. Micro-CT analysis revealed that barrier improvements were influenced not only by porosity but also by network rigidity and diffusion pathway tortuosity introduced by lignin's aromatic structure. These findings demonstrate that lignin-reinforced NIPU films provide a viable route toward fully bio-based, isocyanate-free materials with strong potential for sustainable packaging applications.

## Abstract 2

Conventional polyurethane (PU) films used in flexible packaging commonly rely on petroleum-derived feedstocks and toxic isocyanates. Here, fully bio-based, isocyanate-free thermoplastic films are developed using lignin- and sebacic acid-derived cyclic carbonates.

## Graphical abstract

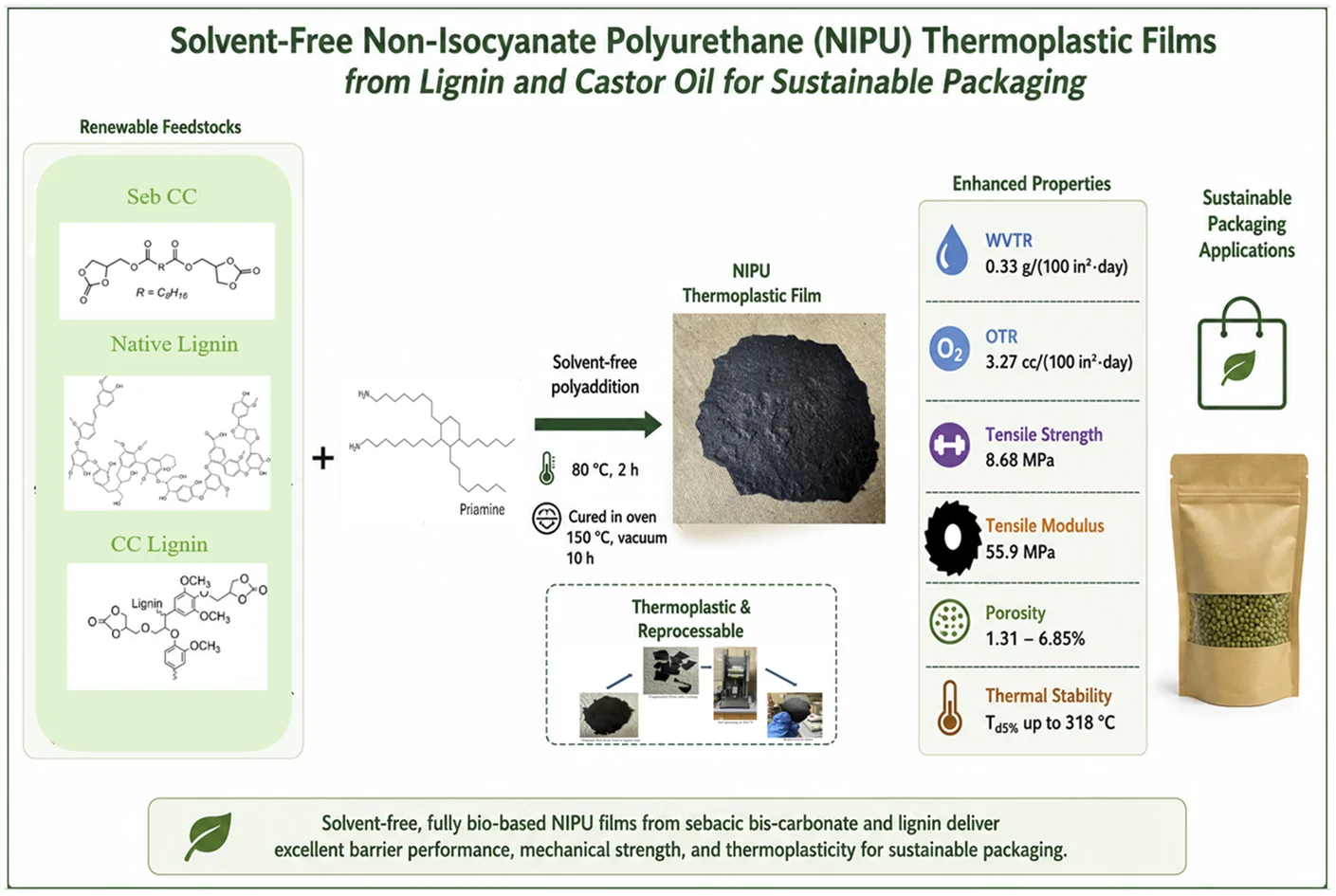

## 1. Introduction

Traditional polyurethane (PU) films, widely used in flexible packaging and coating applications, are mainly prepared from petroleum-based polyols and hazardous isocyanates, resulting in serious environmental and health issues.^1^ In addition to the dependency on fossil-based feedstocks, the production, processing and disposal of isocyanates at the end of their life cycle are associated with serious safety hazards, due to their toxicity and hazardous combustion products.^2^ These issues, combined with an increasing demand for sustainable packaging materials, have driven the development of renewable and isocyanate-free alternatives to polyurethane.

In recent years, among the emerging approaches, non-isocyanate polyurethanes (NIPUs) synthesized *via* cyclic carbonate–amine polyaddition have attracted considerable attention because they avoid the use of toxic isocyanates, while providing the possibility of incorporating renewable feedstocks such as vegetable oils, lignin and biomass-derived monomers.^3,4^ Wang *et al.* synthesized mechanically robust NIPU thermosets from cyclic carbonate linseed oil and Patel *et al.* presented solvent- and catalyst-free soybean oil-based NIPU films with promising thermal and mechanical performance.^3,4^ While these studies demonstrated the feasibility of renewable solvent-free NIPU synthesis, they mainly focused on network formation and bulk mechanical properties, with relatively limited investigations on barrier performance, morphology–transport relationships, and the role of renewable aromatic reinforcement components relevant for sustainable packaging applications. Similarly, Yang *et al.* demonstrated that agricultural residues such as sweet potato waste can be converted into bio-based NIPUs suitable for food preservation applications.^5^ In a subsequent study, the same group reported lignin-oil-derived NIPUs with enhanced ultraviolet resistance and antibacterial properties, highlighting the multifunctional potential of renewable NIPU systems for sustainable packaging applications.^6^ Although these studies highlight the multifunctional potential of bio-based NIPUs, comprehensive evaluation of packaging-relevant properties such as oxygen and moisture barrier performance remains limited.

Lignin has become a very attractive renewable component for polyurethane systems due to its abundant availability, aromatic structure, antioxidant functionality and rigid three-dimensional network containing phenolic and aliphatic hydroxyl groups.^7^ Recently, polyurethanes have been shown to benefit from lignin addition by such properties as increased stiffness, thermal stability, hydrogen bonding interactions and UV resistance. Mahajan *et al.* reported lignin-derived thermoplastic NIPUs with better toughness and hydrogen-bonding content than petroleum-derived analogues.^8^ Gao *et al.* demonstrated lignin-based polyurethane coatings for degradable mulch films. Xue *et al.* demonstrated lignin reinforced polyurethane films exhibit enhanced thermal stability and mechanical stiffness.^9,10^ Wang *et al.* also proved that high modulus polyurethane films can be achieved through the fine-tuning of the lignin content and NCO/OH ratio using lignin-based polyols.^11^ Xue *et al.* comprehensively reviewed lignin-based polyurethane materials and highlighted the importance of lignin structure, hydroxyl content, and compatibility in determining thermal and mechanical performance.^12^ Despite this progress, the majority of high-performance lignin-based polyurethane films reported to date still rely on conventional isocyanate chemistry, and comparatively few studies have systematically investigated lignin-reinforced NIPU films for packaging-relevant applications.^9–12^

In parallel, foundational work by Duval *et al.* and Carré *et al.* established important principles for renewable NIPU synthesis under solvent-free, isocyanate-free, and low-toxicity processing conditions.^13,14^ Duval *et al.* demonstrated organocatalytic synthesis of renewable polyhydroxyurethanes *via* cyclic carbonate ring-opening reactions, while Carré *et al.* reported solvent- and catalyst-free biobased NIPUs with favorable rheological and thermal behavior.^13,14^ These studies confirmed the feasibility of scalable and sustainable NIPU processing; however, detailed evaluation of morphology, permeability, and structure–transport relationships relevant to packaging films was not extensively explored. In particular, the role of lignin chemistry in governing film morphology, porosity, and barrier performance in solvent-free NIPU systems remains poorly understood.

Recent studies on lignin-based NIPU foams have further demonstrated that lignin functionalization can strongly influence network formation and mechanical behavior in isocyanate-free polyurethane systems.^15,16^ However, foam systems fundamentally differ from dense film structures because cellular architectures dominate transport and mechanical behavior in foams, whereas packaging films require continuous dense morphologies capable of minimizing gas and moisture permeation. Consequently, the influence of lignin chemistry on film morphology, porosity, mechanical integrity, and barrier performance in dense NIPU films remains insufficiently understood.

To bridge these gaps, in the present study, fully bio-based, solvent free NIPU films prepared from the castor-oil derived cyclic carbonate, sebacic bis-cyclocarbonate (SebCC), and lignin as a renewable aromatic reinforcement component, were studied. Unlike previous studies that investigated single lignin chemistries, we here directly compare cyclocarbonated lignin (CC lignin) and native lignin (NL) at the same loading to evaluate the effect of lignin functionalization on film formation, morphology, thermal behavior, mechanical performance, permeability and internal porosity. While the long aliphatic backbone of SebCC allows for flexibility and processability, lignin offers aromatic rigidity and intermolecular interactions that can boost barrier performance. The films were prepared *via* solvent free method using fatty acid based dimer diamine with different SebCC : lignin ratio from 1 : 0 to 1 : 1.

The synthesized NIPU films were characterized by FTIR, solid-state ^13^C CP/MAS NMR, DSC, TGA, tensile analysis, water vapor transmission rate (WVTR), oxygen transmission rate (OTR), SEM and micro-computed tomography (micro-CT) to establish comprehensive structure–property relationships important for sustainable packaging applications. This work offers insights into how lignin chemistry dictates network structure, porosity and barrier performance of dense thermoplastic NIPU films by direct comparison of CC lignin with native lignin systems and identifies promising processing strategies for next generation sustainable packaging materials.

## 2. Materials and methods

### 2.1 Materials

Kraft lignin for this study was supplied by Ingevity Corporation (Fort Mill, SC, USA) under the trade name “Indulin AT”. Sebacoyl chloride (97%) was purchased from Alfa Aesar (Karls-ruhe, Germany). Glycerol carbonate (Jeffsol GC, 93%) was obtained from Huntsman (Everberg, Belgium). Dimer diamines (DDA) commercially available under the trade name Priamine was purchased from CRODA (Goole, England). Glycerol carbonate (GC) was purchased from Spectrum Chemical (New Brunswick, NJ, USA) or InKemia Green Chemicals (Houston, TX, USA) with a minimum purity of 90%. Dimethyl carbonate (DMC, >99.0%), 1,5,7-triazabicyclo[4.40]dec-5-ene (TBD, 98%), was purchased from Millipore Sigma (St Louis, MO, USA).

### 2.2 Preparation of cyclocarbonated lignin and sebacic bis-cyclocarbonate

Cyclocarbonated lignin (CC lignin) and sebacic bis-cyclocarbonate (Seb CC) were synthesized according to procedures reported previously by Evancho *et al.*^16^ Briefly, kraft lignin was first functionalized using glycerol carbonate in a stainless-steel Parr reactor under nitrogen at 145 °C to introduce glycerol-derived functionalities. The resulting lignin intermediate subsequently reacted with dimethyl carbonate in the presence of potassium carbonate to generate cyclic carbonate groups, producing cyclocarbonated lignin suitable for non-isocyanate polyurethane synthesis. The product was purified through repeated acidified-water washing, filtration, and vacuum drying prior to use.

Seb CC was synthesized following the procedure reported by Carré *et al.*^14^ and previously employed in our lignin-based NIPU foam study.^16^ Briefly, sebacoyl chloride was reacted with glycerol carbonate in dichloromethane using triethylamine as an acid scavenger under an inert atmosphere. Following reaction, the resulting triethylammonium chloride salt was removed by filtration, and the organic phase was washed, dried, and concentrated under reduced pressure to obtain SebCC as a white solid.

### 2.3 Synthesis of NIPU thermoplastic

The formulation design for the non-isocyanate polyurethane (NIPU) films was adapted from the systematic approach reported by Xue *et al.*^10^ In that study, the authors maintained a fixed NCO/OH ratio while varying the relative proportions of lignin and polyol to evaluate structure–property relationships. Following a similar rationale, the present work maintains a constant cyclic-carbonate-to-amine (CC : amine) molar ratio of 1.0, while adjusting the weight ratios of SebCC to lignin (either CC lignin or native lignin). The comparative formulation matrix for both systems (Seb CC + CC lignin and SebCC + native lignin) is summarized in Table 1, where the Seb CC : lignin ratios range from 1 : 0.25 to 1 : 1 with fixed Seb CC loading (1 g) and stoichiometrically balanced priamine content (Table 1). This design allows a direct and quantitative evaluation of how lignin addition as a filler and cross-linker influences NIPU film performance under equivalent conditions. The formulations were prepared by mixing the respective lignin with Seb CC and curing agent (priamine) in stoichiometric balance with the cyclic carbonate groups. The polyaddition reaction was carried out under a nitrogen atmosphere at 80 °C for 2 hours using TBD as a catalyst. The prepolymer mixtures were then cast and post-cured at 150 °C under vacuum for 10 hours to enhance cross-linking density, thereby improving the thermal and mechanical properties of the resulting films (Fig. 1).

**Table 1:** Formulation matrix for NIPU films prepared with SebCC and CC- or native lignin at varying SebCC : lignin weight ratios, maintaining a constant cyclic-carbonate–to–amine (CC : amine) ratio of 1.0

System	Sample	Seb CC : lignin (w/w)	Priamine (ml)	CC : amine ratio
Only Seb CC	NIPU-Seb CC	1 : 0	1.1	1
Seb CC + CC lignin	NIPU-CC-1	1 : 0.25	1.26	1
Seb CC + CC lignin	NIPU-CC-2	1 : 0.5	1.4	1
Seb CC + CC lignin	NIPU-CC-3	1 : 0.75	1.53	1
Seb CC + CC lignin	NIPU-CC-4	1 : 1	1.66	1
Seb CC + native lignin	NIPU-NL-1	1 : 0.25	1.1	1
Seb CC + native lignin	NIPU-NL-2	1 : 0.5	1.1	1
Seb CC + native lignin	NIPU-NL-3	1 : 0.75	1.1	1
Seb CC + native lignin	NIPU-NL-4	1 : 1	1.1	1

**Fig. 1:**
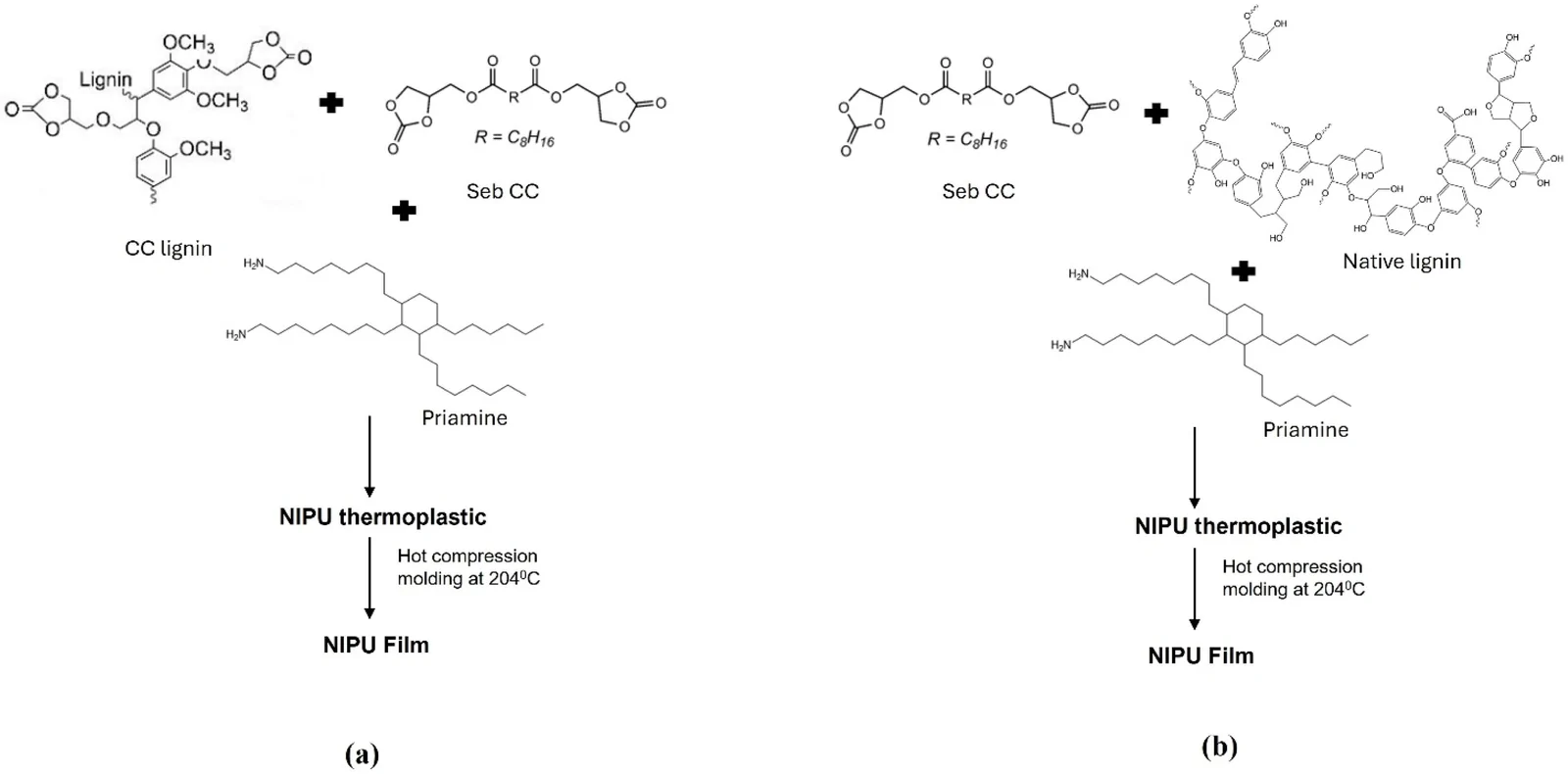
Reaction scheme for CC lignin and native lignin NIPU film systems.

### 2.4 Film processing

To prepare the polyurethane films, hot compression molding was performed using a Carver Laboratory Press (Model 3925, Carver, Inc., Wabash, IN, USA). Initially, the polyurethane was cured within Teflon. After curing, the polyurethane samples were removed from the Teflon molds and placed between release papers to facilitate easy demolding. The compression molding was carried out at 204 °C under an applied load of 8000 lb for 90 s. The use of release paper aided in the clean release of the films from the hot plates, and the resulting films were cooled to ambient temperature before further characterization (Fig. 1).

## 3. Characterization techniques

The characterization of cyclocarbonated lignin (CC lignin) and Seb CC used in this work were previously established in detail in our earlier lignin-based NIPU foam study.^16^ Briefly, successful cyclocarbonation was confirmed by the appearance of characteristic peaks for oxygenated units corresponding to the cyclocarbonate rings in C-NMR. In CC lignin a decrease in signal associated with gamma hydroxyls after lignin functionalization was observed in 2D NMR. Fourier-transform infrared (FTIR) spectroscopy was performed on all film formulations. Infrared spectra were collected on Thermo Nicolet iS10 from 500–4000 cm^−1^ using 16 scans with a spectral resolution of 4 cm^−1^. Peaks associated with urethane formation and aromatic lignin functionalities were analyzed to confirm successful synthesis and incorporation. Differential Scanning Calorimetry (DSC) was done on TA Instruments DSC250 to determine thermal transitions of the cured films, particularly the glass transition temperature (*T*_g_), which indicates polymer rigidity. Thermogravimetric analysis (TGA) was conducted using TA Instruments 2950 TGA, with samples heated from room temperature to 600 °C at a rate of 10 °C min^−1^ under a nitrogen atmosphere to evaluate thermal stability. Mechanical performance was evaluated *via* tensile testing across five trials. Results indicated a trend of increasing tensile strength and modulus with higher lignin content.

Water vapor transmission rates (WVTR) were evaluated using the PERMATRAN-W® Water Vapor Permeability Analyzer (MOCON®, AMETEK®) and were used to assess barrier performance of the films—key attributes for packaging materials. Scanning Electron Microscopy (SEM) analysis was performed on Hitachi 3400. Samples were coated with platinum prior imaging and subsequently the surface morphology of the films were studied. Micro-computed tomography (Micro-CT) was performed using a Rigaku nano3DX X-ray CT scanner equipped with a copper anode X-ray source to evaluate the internal porosity of the NIPU films. Imaging was conducted using a 20× objective with an isotropic voxel size of approximately 188 nm. The reconstructed datasets were analyzed using Dragonfly software to quantify film porosity.

## 4. Results and discussion

Visual observation of the films shows that there are clear differences in surface texture, uniformity and optical clarity as a function of lignin type and loading. The control film composed solely of Seb CC is light in color and relatively transparent, indicating a homogeneous matrix (Fig. 2). The addition of lignin makes both CC lignin and native lignin films darker with higher lignin content, which shows the intrinsic chromophoric properties of lignin. Films with CC lignin seem to be smoother, more uniform and better integrated at higher lignin loadings, especially for NIPU-CC-3 and NIPU-CC-4, indicating good dispersion and compatibility with the matrix. Conversely, native lignin films are more irregular and heterogeneous on the surface, with an evident patchiness and rougher texture at higher loadings, because of poor filler–matrix interaction and sub-optimal dispersion.

**Fig. 2:**
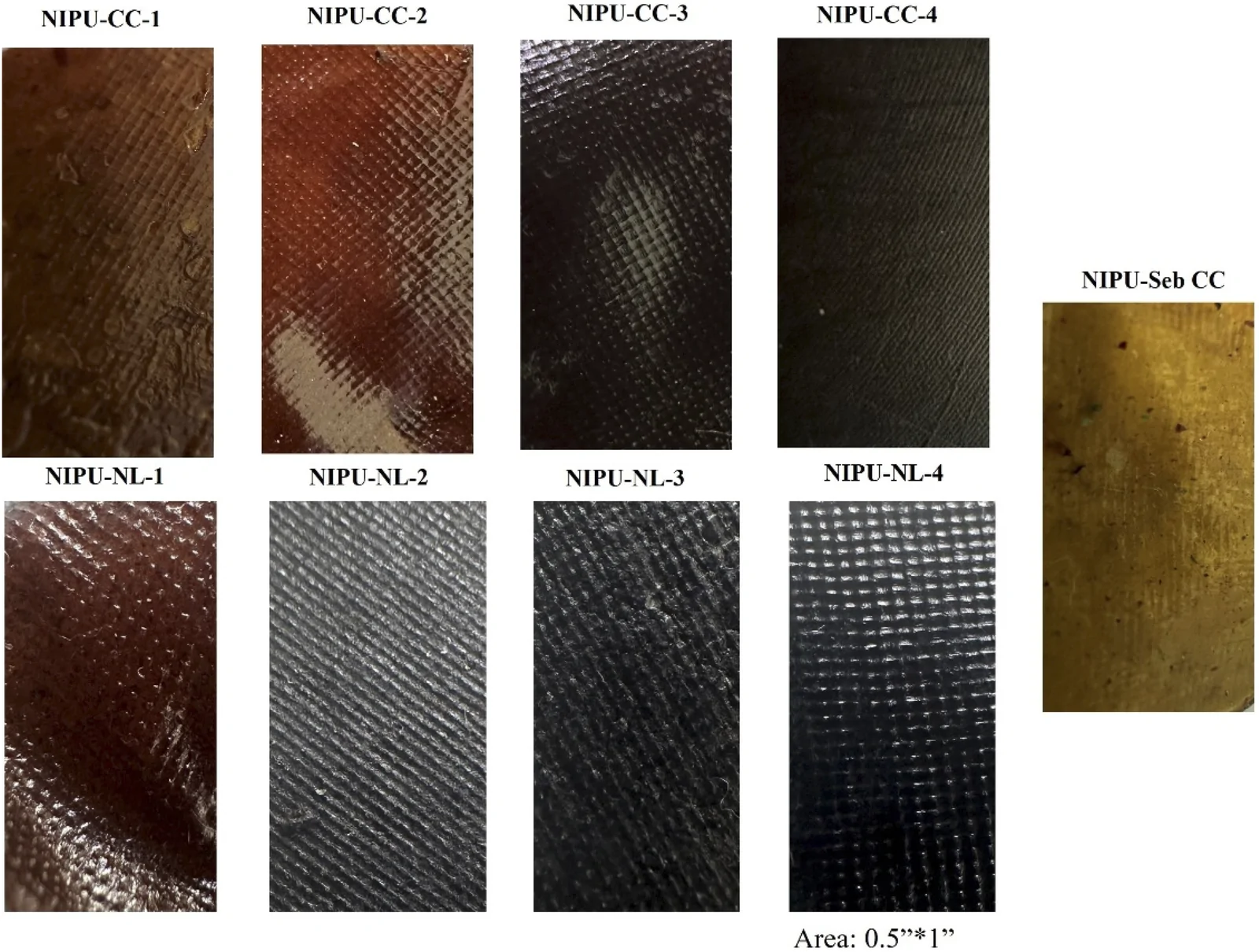
Optical images of the cured CC lignin and native lignin NIPU film systems.

FTIR spectroscopy was used to analyze the chemical structure and verify the formation of non-isocyanate polyurethane (NIPU) films synthesized from sebacic acid biscyclocarbonate (Seb CC) and priamine, incorporating either cyclocarbonated lignin (CC lignin) or native lignin at varying loadings. To more clearly confirm NIPU formation, comparative FTIR spectra of Seb CC, CC lignin, native lignin, and representative NIPU films were analyzed (Fig. 3). In both lignin systems, successful ring-opening polyaddition between cyclic carbonate and amine functionalities is supported by the disappearance of the cyclic carbonate absorption near 1790 cm^−1^ after reaction, together with the appearance of broad N–H stretching vibrations in the 3300–3400 cm^−1^ region and C=O stretching bands near 1700–1720 cm^−1^, characteristic of urethane carbonyls. The presence of the amide II band at ∼1550 cm^−1^ further supports urethane bond formation across all trials.

**Fig. 3:**
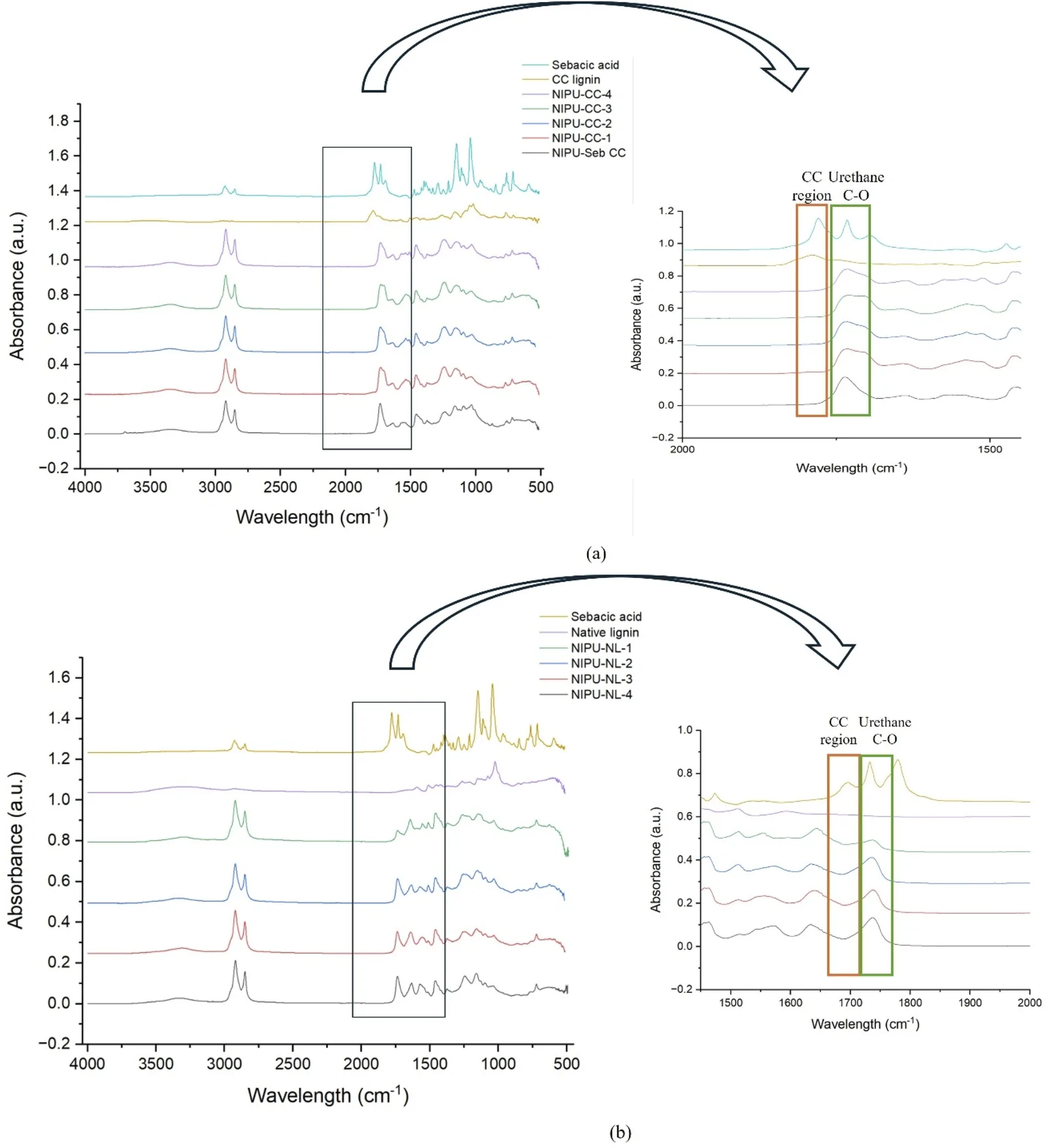
FTIR of NIPU films (a) CC lignin systems. (b) Native lignin systems.

In the case of the CC lignin-based films, increasing the amount of lignin caused the aromatic C=C stretching vibrations in the range of 1510–1600 cm^−1^ to be stronger and the O–H/N–H region to be broader, implying higher aromatic content and stronger hydrogen-bonding interactions in the polymer network. The control film of the Seb CC showed a relatively sharp ester C=O band at around 1730 cm^−1^ and well-defined bands attributed to C–O stretching vibrations in the 1200–1300 cm^−1^ region. Inclusion of lignin made these regions broader and less well defined, indicating that the chemical environment was becoming more heterogeneous owing to the complex aromatic structure and intermolecular interactions of lignin.

Comparative analysis of CC lignin and native lignin systems further emphasizes differences in network formation. The CC lignin films exhibit more pronounced and distinct bands for ether- and carbonate-associated C–O–C in the 1100–1300 cm^−1^ range, indicative of the presence of reactive cyclic carbonate groups that can participate in network formation with priamine. In contrast, the native lignin films exhibit broader and more diffuse carbonyl and C–O bands, which is indicative of the lack of reactive cyclic carbonate groups and the increased contribution of physical aromatic reinforcement and hydrogen-bonding interactions, rather than direct covalent integration into the NIPU network. The results of solid-state ^13^C CP/MAS NMR further confirm the above FTIR observations and confirm the evolution of carbon environments associated with urethane and the lignin-rich polymer network structures after reaction.

These FTIR observations are further supported by solid-state ^13^C CP/MAS NMR analysis (Fig. 4), which provides detailed insight into the carbon environments and network alkyl chains, confirming the predominantly flexible aliphatic nature of the base NIPU matrix. A weaker feature near 15–20 ppm is attributed to terminal –CH_3_ groups in the fatty-acid-derived segments. With increasing lignin incorporation, the –CH_2_– envelope becomes progressively broadened, reflecting reduced segmental mobility and increased network constraint in lignin-reinforced systems. A distinct signal at ∼55–56 ppm arises from methoxy (OCH_3_) groups characteristic of lignin, providing a direct spectral marker of lignin incorporation. The intensity of this band increases systematically with lignin loading and is most pronounced in native lignin films, consistent with their higher methoxy content. The broadened region between 60 and 90 ppm corresponds to C–O-rich environments, including carbons adjacent to hydroxyl and ether functionalities in ring-opened carbonate structures and lignin side chains, reflecting increased heterogeneity and polar functionality in lignin-containing networks.^15,17^

**Fig. 4:**
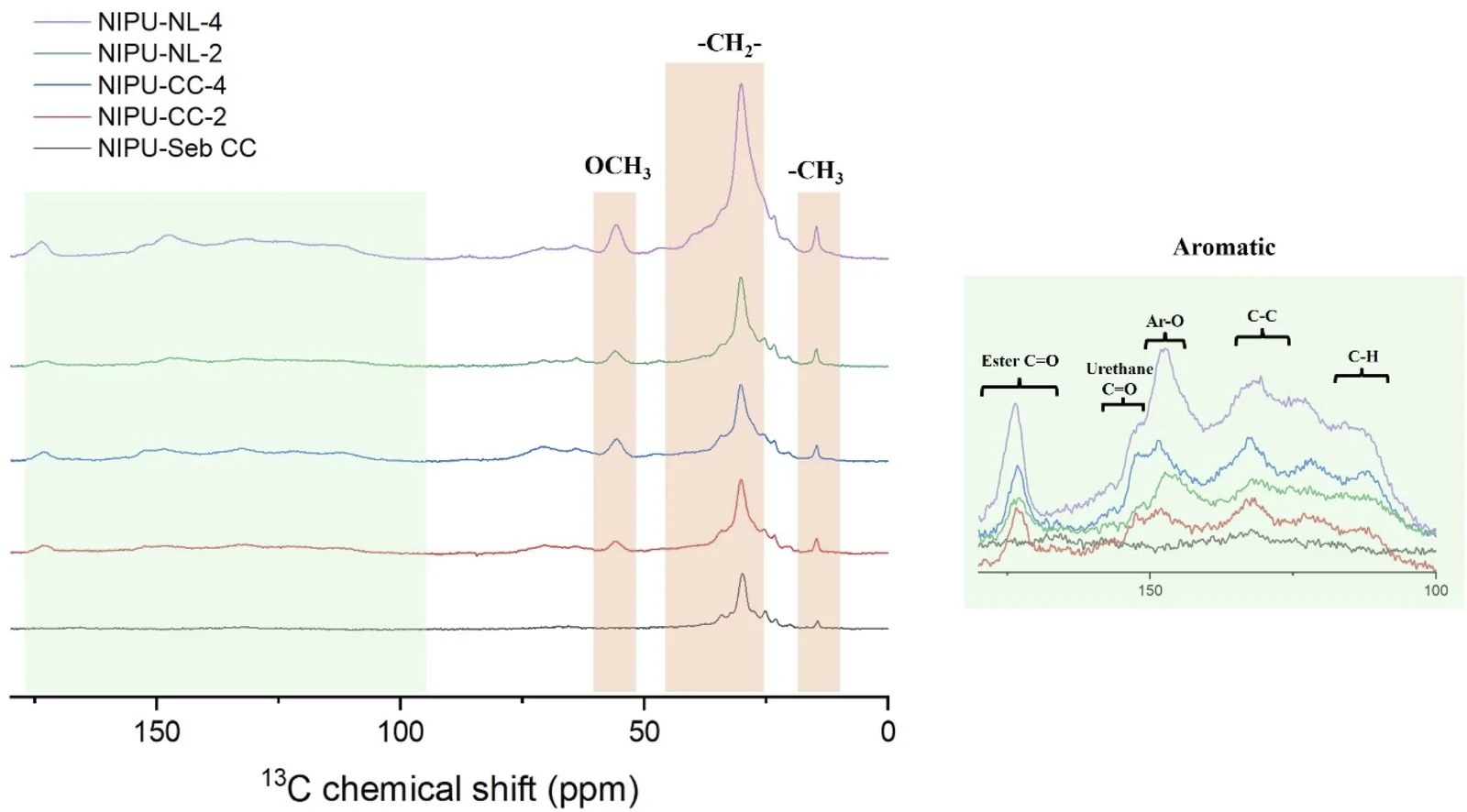
^13^C CP/MAS NMR at 13 kHz of NIPU films.

With the addition of lignin, there is a pronounced evolution in the aromatic region (100–160 ppm) that can be divided into chemically different domains. The 100–120 ppm region assigned to protonated aromatic C–H carbons, confirming the presence of intact lignin aromatic rings. The intermediate region at 120–145 ppm is assigned to substituted aromatic C–C carbons of the lignin backbone and interunit linkages, with progressive broadening indicating increasing heterogeneity and rigid domain formation. Most importantly, the 145–160 ppm region features overlapping contributions from oxygen-substituted aromatic Ar–O carbons and urethane (carbamate) carbonyls, which directly provide evidence for the formation of urethanes within lignin-rich aromatic domains. The increase in this region with increasing lignin loading, especially in native lignin systems, indicates increased hydrogen bonding, aromatic stacking and chemical incorporation of urethane linkages into rigid aromatic environments. In the high-frequency region, resonances observed between 170 and 175 ppm are assigned to ester carbonyl carbons originating from the sebacic backbone. These ester carbonyl signals are weak or barely detectable in the pure Seb CC film, reflecting the high mobility of the aliphatic matrix and limited cross-polarization efficiency. With lignin incorporation, this region becomes progressively more visible, indicating reduced segmental mobility and stabilization of ester carbonyl environments within rigid aromatic-rich networks. Overall, the concerted evolution of aliphatic architecture of the NIPU films. All samples exhibit a dominant resonance in the 28–35 ppm region, assigned to aliphatic –CH_2_– carbons from the sebacic acid backbone and priamine methoxy, aromatic, urethane, and ester carbonyl regions confirms progressive lignin incorporation, formation of chemically integrated urethane–aromatic domains, and network rigidification, with distinct differences in chemical integration and chain mobility between CC-lignin- and native-lignin-reinforced NIPU films.^15,17^

The glass transition temperature (*T*_g_) of NIPU films increased with lignin incorporation in both systems, indicating reduced segmental mobility due to enhanced structural rigidity. In the CC lignin-based films, *T*_g_ rose steadily from −19.69 °C (NIPU-Seb CC) to −5.11 °C for NIPU-CC-4, with intermediate values of −11.77 °C, −6.86 °C, and −7.52 °C corresponding to NIPU-CC-1, NIPU-CC-2, NIPU-CC-3, respectively (Fig. 5 and Table 2). This progressive trend suggests effective chemical incorporation of CC lignin into the polyurethane network, likely due to the presence of reactive cyclic carbonate groups that enable covalent bonding with amine curing agents. The more uniform integration of CC lignin promotes consistent crosslinking and limits polymer chain flexibility, leading to a gradual increase in *T*_g_ with increasing lignin content.

**Fig. 5:**
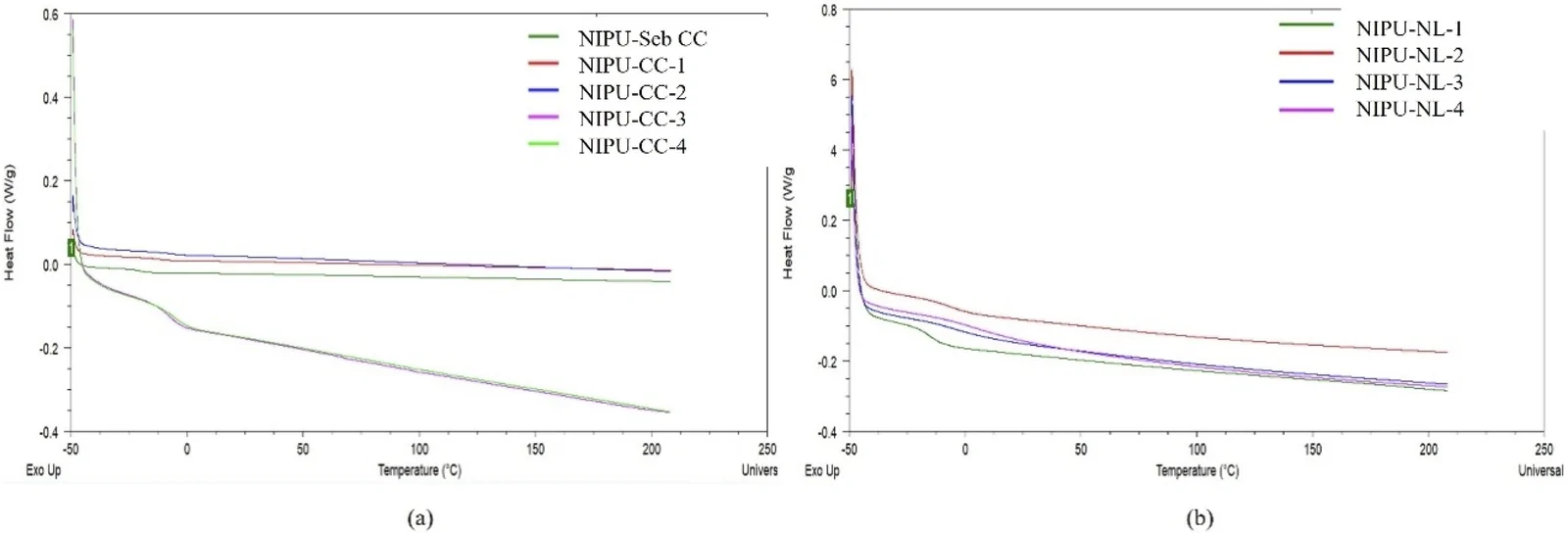
DSC of NIPU films (a) CC lignin trials. (b) Native lignin trials.

**Table 2:** Glass transition temperature (*T*_g_), weight loss (*T*_d5%_), and maximum degradation temperature (*T*_max_) of NIPU films of CC lignin and native lignin systems

NIPU system	*T*_g_ (°C)	*T*_d5%_	*T*_max_
NIPU-Seb CC	−19.69	277	459.2
NIPU-CC-1	−11.77	287	459.6
NIPU-CC-2	−6.86	306	457.5
NIPU-CC-3	−7.52	267	451.5
NIPU-CC-4	−5.11	294	447.6
NIPU-NL-1	−15.62	263	450
NIPU-NL-2	−6.70	318	447
NIPU-NL-3	−4.25	306	447
NIPU-NL-4	3.78	346	440

In contrast, native lignin-based films displayed a sharper *T*_g_ increase, particularly at higher lignin loadings—from −19.69 °C to 3.78 °C as lignin content increased from NIPU-NL-1 to NIPU-NL-4. While the initial rise in *T*_g_ (−15.62 °C for NIPU-NL-1) reflects lignin's inherent rigidity, the steep transitions for NIPU-NL-2 (−6.70 °C), NIPU-NL-3 (−4.25 °C), and NIPU-NL-4 (3.78 °C) likely stem from physical rather than chemical interactions (Fig. 5 and Table 2). Native lignin, lacking reactive cyclic carbonates, is not covalently bonded into the network and may exhibit poor dispersion at higher concentrations. This can lead to phase-separated lignin-rich domains that stiffen the surrounding matrix, a phenomenon known as microdomain stiffening. These rigid microdomains restrict local chain mobility, resulting in a pronounced increase in *T*_g_. However, this behavior also suggests heterogeneity in network structure and possible compromises in material processability and uniformity at higher native lignin loadings.

Thermogravimetric analysis (TGA) was used to evaluate the thermal stability of NIPU films by measuring the temperature at 5% weight loss (*T*_d5%_), and maximum degradation temperature (*T*_max_). These parameters help assess the onset and progression of thermal degradation as a function of lignin type and loading for both CC lignin- and native lignin-based NIPU films (Fig. 6 and Table 2). In the CC lignin series, incorporating CC lignin significantly enhanced the thermal degradation resistance of the films, with *T*_d5%_ increasing from 277 °C in the lignin-free control to 287 °C and a peak of 306 °C, respectively. This improvement is attributed to the aromatic structure and possible hydrogen bonding interactions from CC lignin, which stabilize the polymer network and delay thermal decomposition. *T*_max_ values remained high and relatively stable (459.6 °C and 457.5 °C), further supporting the role of CC lignin in enhancing thermal resistance. However, for trials NIPU-CC-3 and NIPU-CC-4 respectively, *T*_d5%_ declined to 267 °C and recovered slightly to 294 °C, while *T*_max_ decreased to 451.5 °C and 447.6 °C, respectively. This slight drop in thermal performance at higher loadings could result from over-saturation, reduced crosslinking density, or localized phase separation, which disrupts the uniformity of the degradation pathway.

**Fig. 6:**
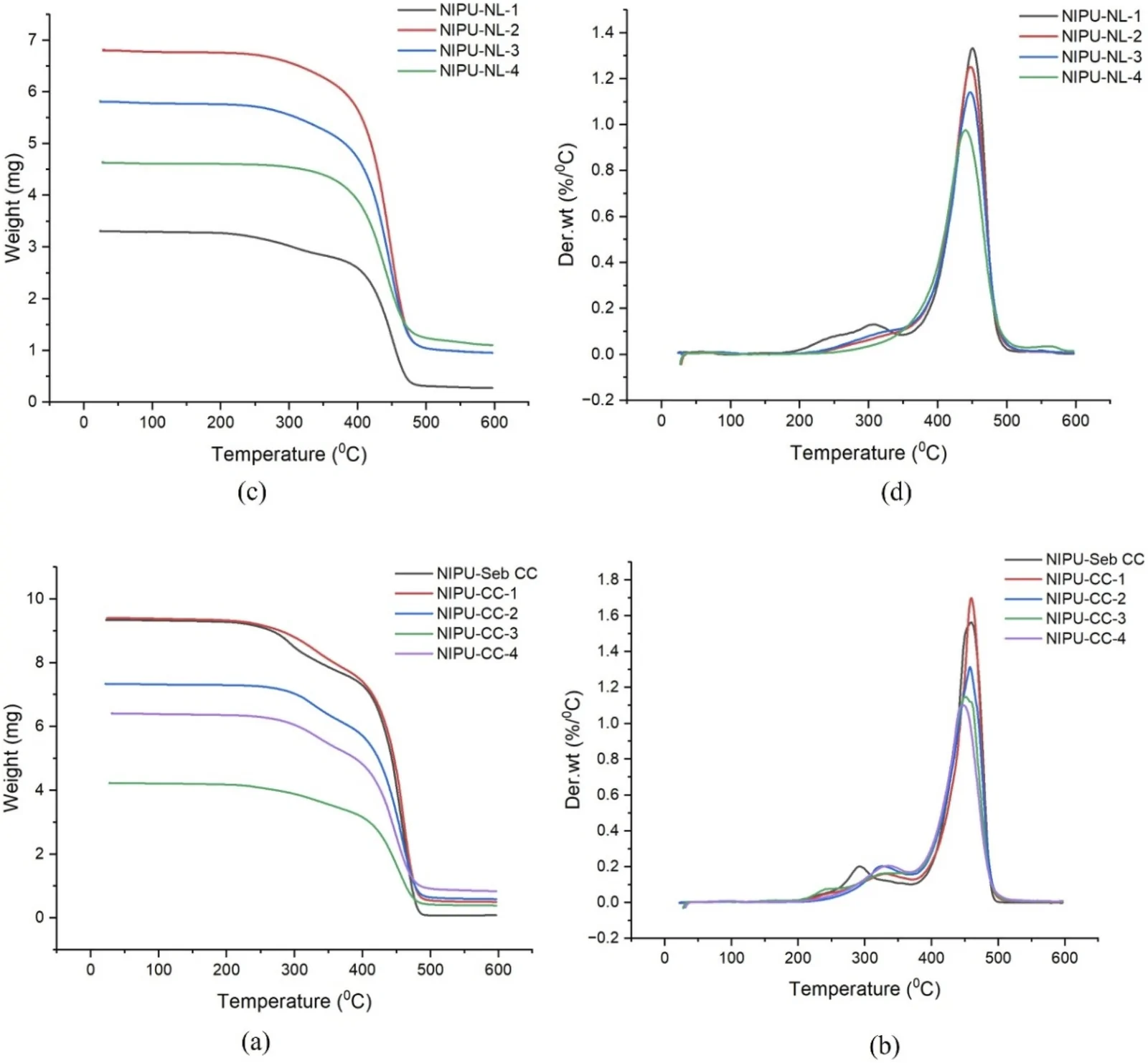
(a and b) TGA and DTG curves for CC lignin systems compared to control (Seb CC only). (c and d) TGA and DTG curves for native lignin systems.

In contrast, native lignin films displayed a different thermal behavior. At low loading, *T*_d5%_ dropped to 263 °C—lower than NIPU-Seb CC—indicating poor thermal reinforcement due to limited interfacial interaction and dispersion. However, increasing native lignin content significantly improved *T*_d5%_ to 318 °C and 306 °C, respectively, surpassing all CC lignin formulations. Notably, NIPU-NL-4 yielded the highest *T*_d5%_ of 346 °C, demonstrating delayed onset of degradation likely due to lignin's high thermal resistance and its accumulation in the matrix. Nonetheless, *T*_max_ consistently decreased across native lignin films, from 459.2 °C in NIPU-Seb CC to 440 °C, suggesting that while native lignin may delay the initial breakdown, it promotes faster bulk decomposition at elevated temperatures. This divergence implies that although native lignin can reinforce initial degradation thresholds at high loadings, its poor dispersion and structural heterogeneity limit its efficiency in sustaining thermal stability during peak degradation.

The incorporation of lignin produced clear and contrasting mechanical trends between the CC lignin and native lignin NIPU systems (Fig. 7 and Table 3). The ultimate tensile strength of the CC lignin series increased steadily from 0.37 MPa for NIPU-Seb CC to a maximum of 8.59 MPa at NIPU-CC-3, and then slightly decreased to 6.18 MPa for the highest loading (NIPU-CC-4). The tensile modulus also increased with a similar reinforcement trend from 0.64 MPa to 39.48 MPa at NIPU-CC-4, whereas the elongation at break decreased from 70 to 14% in the series. These results suggest that CC lignin is involved in network construction through reactive cyclic carbonate functionalities that can undergo ring-opening reactions with priamine, leading to better network incorporation, higher stiffness, and lower chain mobility. The small decrease in strength and elongation at the highest loading is probably caused by the increased heterogeneity and stress concentrations related to lignin-rich domains.

**Fig. 7:**
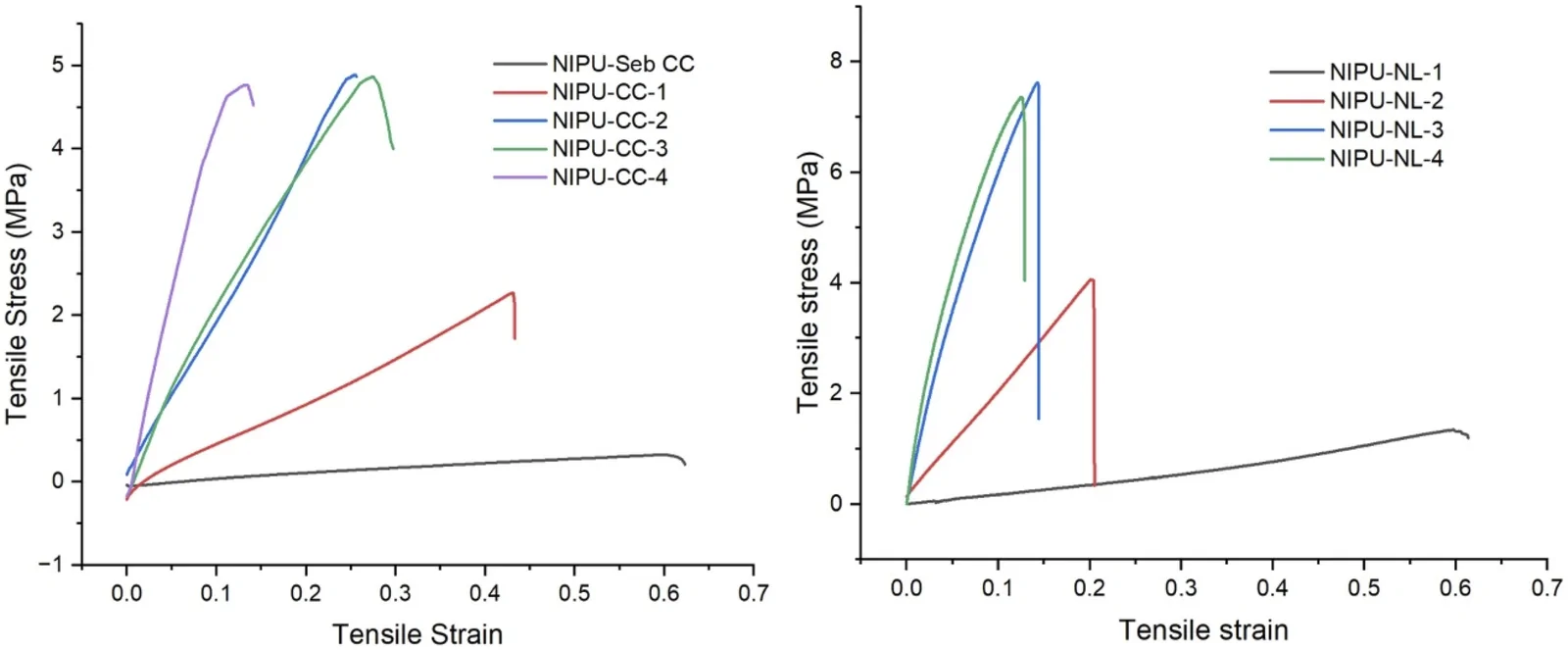
Tensile stress–strain behavior of (a) CC lignin and (b) native lignin systems.

**Table 3:** Mechanical properties of NIPU film systems

NIPU system	Ultimate tensile strength (MPa)	Elongation at break (%)	Tensile modulus (MPa)
NIPU-Seb CC	0.37 ± 0.07	70 ± 15.15	0.64 ± 0.24
NIPU-CC-1	2.71 ± 0.43	51 ± 6.4	5 ± 1.27
NIPU-CC-2	5.68 ± 1.37	27 ± 7.7	21.96 ± 5.77
NIPU-CC-3	8.59 ± 2.5	28 ± 4.62	24.5 ± 5.78
NIPU-CC-4	6.18 ± 1.58	14 ± 3.37	39.48 ± 7.59
NIPU-NL-1	1.52 ± 0.27	68.2 ± 11.5	2.27 ± 0.6
NIPU-NL-2	4.75 ± 1.68	23.7 ± 3.2	15.25 ± 3.51
NIPU-NL-3	8.68 ± 0.71	17.38 ± 2.8	48.76 ± 4.2
NIPU-NL-4	8.15 ± 1.71	12.03 ± 0.2	64.5 ± 9.03

Native lignin films displayed a different reinforcement mechanism, mostly driven by physical interactions. The UTS was between 1.52 MPa (NIPU-NL-1) and 8.68 MPa (NIPU-NL-3) with a slight decrease at the highest loading (8.15 MPa for NIPU-NL-4). The modulus increased dramatically from 2.27 MPa to 64.5 MPa over the series, indicating a significant stiffening effect from the addition of rigid aromatic domains of lignin. This was accompanied by a significant decrease in ductility however, with elongation at break falling from 68.2% to 12.03%. Interestingly, many native lignin formulations had similar or greater tensile strengths than CC lignin counterparts. This observation does not indicate failed cyclocarbonation of lignin but rather represents different reinforcement mechanisms in the two systems. Native lignin, on the other hand, acts as a stiff aromatic reinforcing phase that can limit segmental motion through strong intermolecular interactions, hydrogen bonding and aromatic packing effects. CC lignin, on the other hand, promotes enhanced compatibility and covalent incorporation in the NIPU network. These dense aromatic domains can increase resistance to deformation and contribute to higher tensile strength despite reduced covalent interfacial bonding. This interpretation is consistent with the superior barrier performance, lower effective porosity, and stronger aromatic character observed for the native lignin systems through permeability testing, micro-CT analysis, SEM, and solid-state NMR characterization. Overall, CC lignin provides more homogeneous network integration and balanced reinforcement, whereas native lignin produces greater stiffness and barrier enhancement through rigid aromatic-domain reinforcement.

To evaluate the thermoplastic reprocessability of the developed NIPU films, representative high-lignin formulations containing cyclocarbonated lignin (NIPU-CC-4) and native lignin (NIPU-NL-4) were mechanically fragmented and subsequently reprocessed by hot pressing under the same conditions used during initial film fabrication. As shown in Fig. 8, both formulations were successfully reshaped into continuous, defect-free films after repeated hot pressing without macroscopic cracking or structural failure. The ability of the films to soften upon heating and reform into self-supporting films is attributed to the predominantly amorphous polyhydroxyurethane network, which permits thermoplastic flow during hot pressing while maintaining network integrity upon cooling. The corresponding mechanical properties following successive molding cycles are summarized in Table 4, where the “First” cycle represents the original film prior to any reprocessing, while the “Second” and “Third” cycles correspond to the first and second remolding steps, respectively. Both formulations remained mechanically robust after repeated reprocessing, although a gradual decrease in tensile stress at yield and elongation at break was observed with successive molding cycles, particularly for the native lignin formulation. Such a decrease is likely due to the repeated thermal and mechanical treatment that may induce subtle changes in the chain packing, hydrogen-bonding interactions, and interfacial adhesion of the lignin-filled polymer matrix. Conversely, Young's modulus showed a relatively stable trend and a slight increase after reprocessing, which can be attributed to the fact that the repeated hot pressing resulted in better film consolidation and densification, giving rise to a marginal increase of stiffness, despite the decrease of strength and ductility. In general, the films maintained enough mechanical properties after multiple remolding cycles, confirming the thermoplastic and reprocessable characteristics of the developed lignin-based NIPU systems and their potential compatibility with mechanical recycling for sustainable packaging applications.

**Fig. 8:**
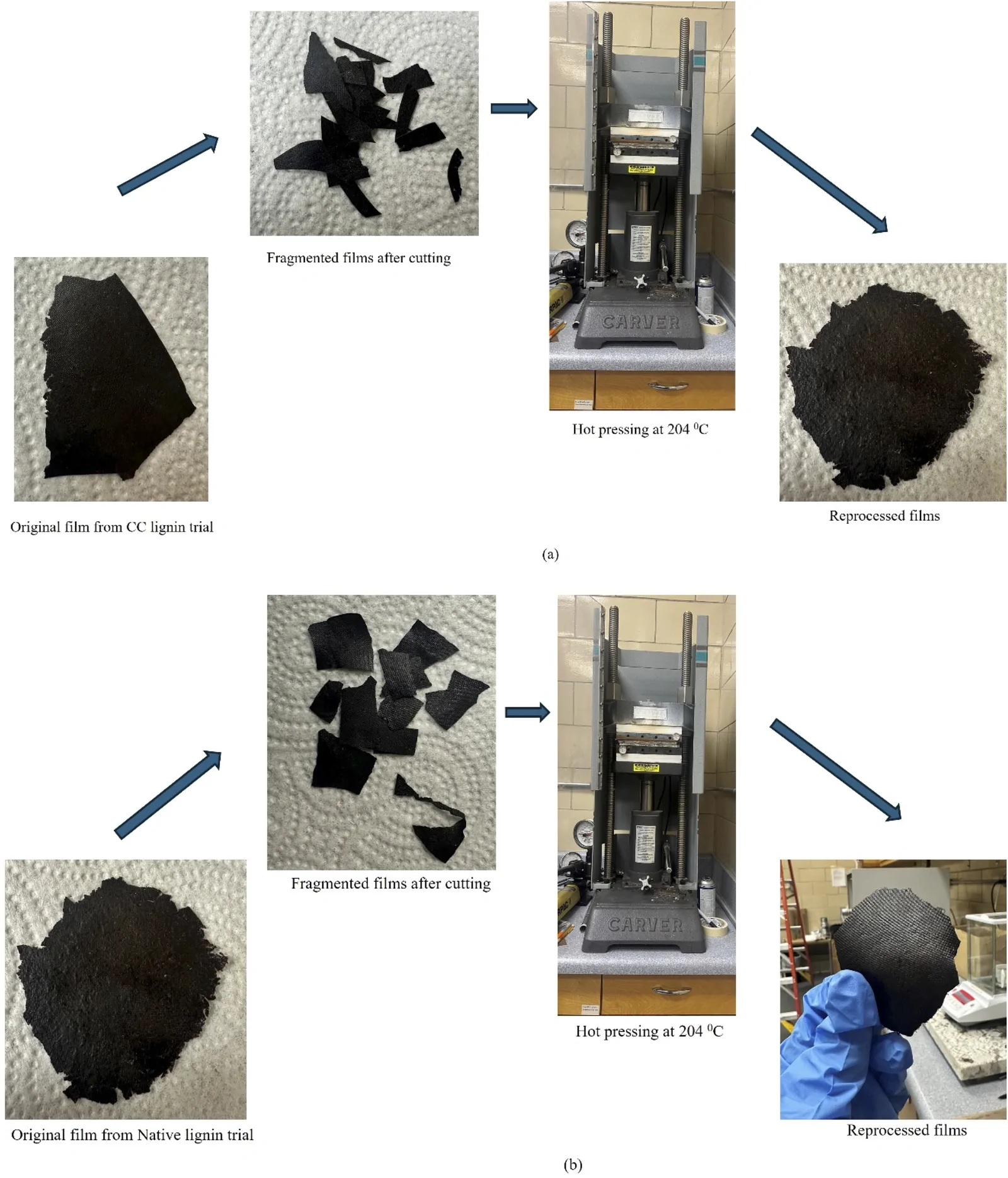
Thermoplastic remolding behavior of representative (a) CC lignin and (b) native lignin NIPU films showing original films, fragmented films after cutting, and reprocessed films obtained after hot-press molding.

**Table 4:** Mechanical properties of representative NIPU films after successive hot-press remolding cycles

Sample	Molding cycle	Tensile stress at yield (MPa)	Young's modulus (MPa)	Elongation at break (%)
NIPU-CC-4	First	4.14 ± 0.2	42.47 ± 2.37	11.89 ± 1.71
	Second	3.51 ± 0.28	39.92 ± 3.32	10.59 ± 1.1
	Third	3.63 ± 0.48	54.5 ± 4.88	9.85 ± 1.75
NIPU-NL-4	First	9.58 ± 0.71	77.36 ± 14.43	12.49 ± 1.17
	Second	6.98 ± 0.64	85.97 ± 6.87	8.21 ± 0.19
	Third	5.53 ± 0.49	86 ± 15.19	7.52 ± 0.85

The non-isocyanate polyurethane (NIPU) films produced in this study show a clear decrease in water-vapor transmission rate (WVTR) with increasing lignin content, for both CC lignin and native lignin systems (Table 5). At 90% RH, the CC lignin series decreased from 5.33 to 1.70 g (100 in^2^·day)^−1^ as the SebCC : CC lignin ratio was reduced from 1 : 0.25 to 1 : 1 (NIPU-CC-1 → NIPU-CC-4). The native lignin NIPUs exhibited an even greater improvement, decreasing from 2.65 to 0.33 g (100 in^2^·day)^−1^ (NIPU-NL-1 → NIPU-NL-4). When benchmarked against biodegradable polymers reported by Wu *et al.*, the lignin-rich NIPUs demonstrate barrier properties that exceed or rival many commercial biopolymers.^18^ For example, PGA, one of the best biodegradable barrier polymers, has a WVTR of ∼0.65 g (100 in^2^·day)^−1^, while PHBV exhibits ∼5.16 g (100 in^2^·day)^−1^. In contrast, widely used biodegradable packaging polymers such as PLA and PBS show much poorer moisture-barrier performance, with WVTR values in the range of 16–39 g (100 in^2^·day)^−1^.

**Table 5:** Water vapor transmission rate (WVTR) and oxygen transmission rate (OTR) of NIPU films of CC and native lignin systems

NIPU system	Water vapor transmission, 100% RH (g (100 in^2^ day)^−1^)	Oxygen transmission, 0% RH (cc (100 in^2^ day)^−1^)	Porosity (%)
NIPU-Seb CC	4.43	39.14	3.10
NIPU-CC-1	5.33	30.68	3.24
NIPU-CC-2	4.02	54.52	6.85
NIPU-CC-3	3.78	42.27	3.60
NIPU-CC-4	1.70	22.29	5.07
NIPU-NL-1	2.65	37.01	1.31
NIPU-NL-2	1.56	14.57	3.44
NIPU-NL-3	0.75	3.27	2.02
NIPU-NL-4	0.33	6.62	3.52

The WVTR values of the lignin-based NIPUs, especially the native lignin formulations (NIPU-NL-3 and NIPU-NL-4), are comparable to those of high-barrier PGA (0.75 and 0.33 g (100 in^2^ day)^−1^) and significantly superior to PHBV, PLA, and PBS against landscape.

The oxygen transmission rates (OTR) of lignin-based NIPU films at 0% RH clearly show a dependence on lignin content and chemistry. Native lignin formulations exhibit OTR values as low as 3.27–6.62 cc (100 in^2^ day)^−1^ (NIPU-NL-3 and NIPU-NL-4), much better than neat NIPU-Seb CC (39.14 cc (100 in^2^·day)^−1^) and lignin-lean systems. Wu *et al.* reported that commonly used biodegradable polymers such as PLA and PBS generally have high oxygen permeability, often >∼13–40 cc (100 in^2^ day)^−1^. PHBV has moderate oxygen-barrier performance of ∼2–5 cc (100 in^2^ day)^−1^, depending on crystallinity and processing conditions.^18^

Micro-CT analysis provides further structural insight. The average porosity of the CC lignin films (≈4–5%) is generally higher than that of the native lignin films (≈2–3%), and this trend broadly aligns with the superior barrier performance observed in the native lignin series. However, the correlation is not strictly linear; samples with comparable total porosity values exhibit markedly different WVTR and OTR results. This indicates that total closed-pore percentage alone does not govern barrier performance. Rather, vapor and gas transport are controlled primarily by pore connectivity, effective tortuosity of diffusion pathways, and the reduction of polymer free volume induced by rigid aromatic domains. In particular, the dense, hydrogen-bonded aromatic network of native lignin increases diffusion path length and restricts segmental mobility, thereby suppressing permeant transport more effectively than CC lignin systems, even when overall porosity values are similar.

The SEM micrographs of the CC lignin and native lignin NIPU films reveal distinct trends in surface morphology that correspond closely to their water-vapor barrier performance (see SI). It should be noted that some small surface bumps and shallow surface features observed across several samples may originate from the compression molding process and contact with the Teflon release paper during film pressing. These features appear as rounded protrusions or shallow impressions and are distributed relatively uniformly across the surface. In contrast, the features interpreted as lignin-related domains or pull-outs typically appear as irregular cavities, voids, or particle-shaped depressions associated with filler dispersion and interfacial interactions within the polymer matrix.

In the CC lignin series, films formulated at Seb CC : CC lignin ratios of 1 : 0.25 and 1 : 0.50 (NIPU-CC-1 and NIPU-CC-2) exhibit relatively smooth and continuous surfaces with only small, finely dispersed particulate domains. This indicates good interfacial compatibility between CC lignin and the polyurethane matrix. As the lignin content increases to a ratio of 1 : 0.75 (NIPU-CC-3), the surface remains largely homogeneous with minimal visible phase separation. Only at the highest loading (NIPU-CC-4, 1 : 1 ratio) does the surface roughness increase noticeably, with more densely distributed lignin-rich microdomains and occasional particle pull-outs visible. These observations suggest effective chemical integration of CC lignin into the polymer network at moderate loadings, while higher loadings introduce localized heterogeneity associated with increased filler content.

In contrast, the native lignin NIPUs show progressively more pronounced morphological heterogeneity as lignin content increases. At 1 : 0.25 and 1 : 0.50 (NIPU-NL-1 and NIPU-NL-2), the surfaces contain dispersed spherical features and occasional shallow cavities that are attributed to lignin particle domains and limited interfacial adhesion between native lignin and the polyurethane matrix. As lignin incorporation increases to 1 : 0.75 and 1 : 1 (NIPU-NL-3 and NIPU-NL-4), larger lignin-rich aggregates and deeper pull-out structures become visible, producing a rougher and more textured morphology. These pull-outs are distinguishable from the pressing-related surface bumps by their irregular shapes and cavity-like appearance, indicating localized detachment of lignin domains from the surrounding polymer matrix.

Despite the increased surface heterogeneity observed in the native lignin systems, these films remain comparatively dense and show fewer interconnected void structures than the CC lignin counterparts. This reflects the rigid aromatic structure of unmodified lignin and its role as a physically reinforcing filler within the NIPU matrix.

These morphological distinctions translate directly to the observed water-vapor and oxygen barrier performance at 90% RH and 0% RH, respectively, as reported above. The lowest-performing native lignin formulation (NIPU-NL-1) already outperforms most CC lignin films, while the highest native lignin formulations (NIPU-NL-3 and NIPU-NL-4) approach the barrier performance of high-barrier biodegradable polymers such as PGA.^18^ In parallel, oxygen-barrier performance follows a similar trend, with lignin-rich native formulations exhibiting markedly reduced OTR values, approaching or matching those reported for PHBV and substantially outperforming PLA and PBS.^18^ This superior barrier behavior—despite more irregular morphology—suggests that the dense aromatic structure and inherent rigidity of native lignin reduce free-volume pathways and lengthen effective diffusion paths, thereby suppressing both moisture and oxygen transport more effectively than CC lignin.

Overall, the CC lignin system exhibits smoother morphology and more uniform dispersion, whereas the native lignin films deliver substantially stronger combined moisture- and oxygen-barrier performance. These observations demonstrate that lignin chemistry, rather than dispersion quality alone, plays a decisive role in governing vapor and gas transport in bio-based NIPU films. Importantly, both CC lignin and native lignin NIPU films maintain barrier performance superior to PLA, PBS, and PHBV, underscoring their potential as high-performance, fully bio-based barrier materials.

## 5. Conclusion

Herein, we demonstrate the successful preparation of NIPU films based on SebCC and Priamine reinforced with CC lignin or native lignin as sustainable bio-aromatic fillers. FTIR analysis confirmed the complete conversion of cyclic carbonates and the formation of urethane linkages for all formulations with increasing presence of aromatic and hydroxyl character at higher lignin incorporations. Thermal analysis revealed that the CC lignin was the most effective for improving the thermal stability at intermediate loadings whereas the native lignin gave rise to the sharpest increase in both *T*_g_ and *T*_d5_% at higher ratios, consistent with restricted chain mobility and emerging microphase separation.

Moreover, the hot-press remolding behavior of CC lignin and native lignin NIPU films indicated their thermoplastic processability and prospective use for recyclable melt-processed packaging applications.

Mechanical testing showed an increase in tensile strength and modulus for both types of lignin as compared to NIPU-Seb CC film. The native lignin with a ratio of 1 : 1 (SebCC : lignin) showed the highest stiffness and strength but with the reduction of elongation, indicating the increased brittleness from the filler aggregation. These trends were confirmed *via* SEM imaging, where CC lignin films were shown to have smooth and homogeneous morphologies with good dispersion, while native lignin films exhibited increasing aggregate sizes and surface heterogeneity with increasing loading levels.

The barrier analysis demonstrated that both lignin systems enhanced water-vapor and oxygen barrier properties with the native lignin NIPUs resulting in the most significant improvements. The WVTR values for the native lignin series ranged from 2.65 to 0.33 g (100 in^2^ day)^−1^ which were better than the CC lignin films and common biodegradable packaging polymers such as PLA, PBS and PHBV. The oxygen transport was similarly affected, with OTR values as low as 3.27–6.62 cc (100 in^2^·day)^−1^ for native lignin-rich formulations, approaching and even matching the oxygen-barrier performance of PHBV. The results suggest that the dense aromatic structure of native lignin, even with less uniform dispersion, is more effective in hindering both moisture and oxygen transport than CC lignin at similar loadings.

In summary, the work highlights the complementary benefits of both types of lignin, with CC lignin offering better dispersion and mechanical uniformity, and native lignin showing excellent moisture barrier properties comparable to or even exceeding those of widely used biodegradable polymers. These results altogether show that lignin reinforced NIPUs are a promising route towards high performance, renewable and solvent free polyurethane films for sustainable packaging applications.

## Conflicts of interest

The authors declare no competing interests.

## Supplementary Material

RA-016-D6RA02212J-s001

## Data Availability

The data supporting this study are available within the article. Additional data related to this work are available from the corresponding author upon reasonable request. Supplementary information: the SEM images of NIPU films. See DOI: https://doi.org/10.1039/d6ra02212j.
