# Supplementary material for: Solvent-free non-isocyanate polyurethane thermoplastic films from lignin and castor oil: toward sustainable packaging applications
Source: RSC Adv. 2026 Sep 11;16(44):54724–37. doi: 10.1039/d6ra02212j (PMC13566744; doi:10.1039/d6ra02212j)
Supplement: Supplementary file 1 [file RA-016-D6RA02212J-s001.pdf]

Supplementary Information (SI) for RSC Advances.
This journal is © The Royal Society of Chemistry 2026

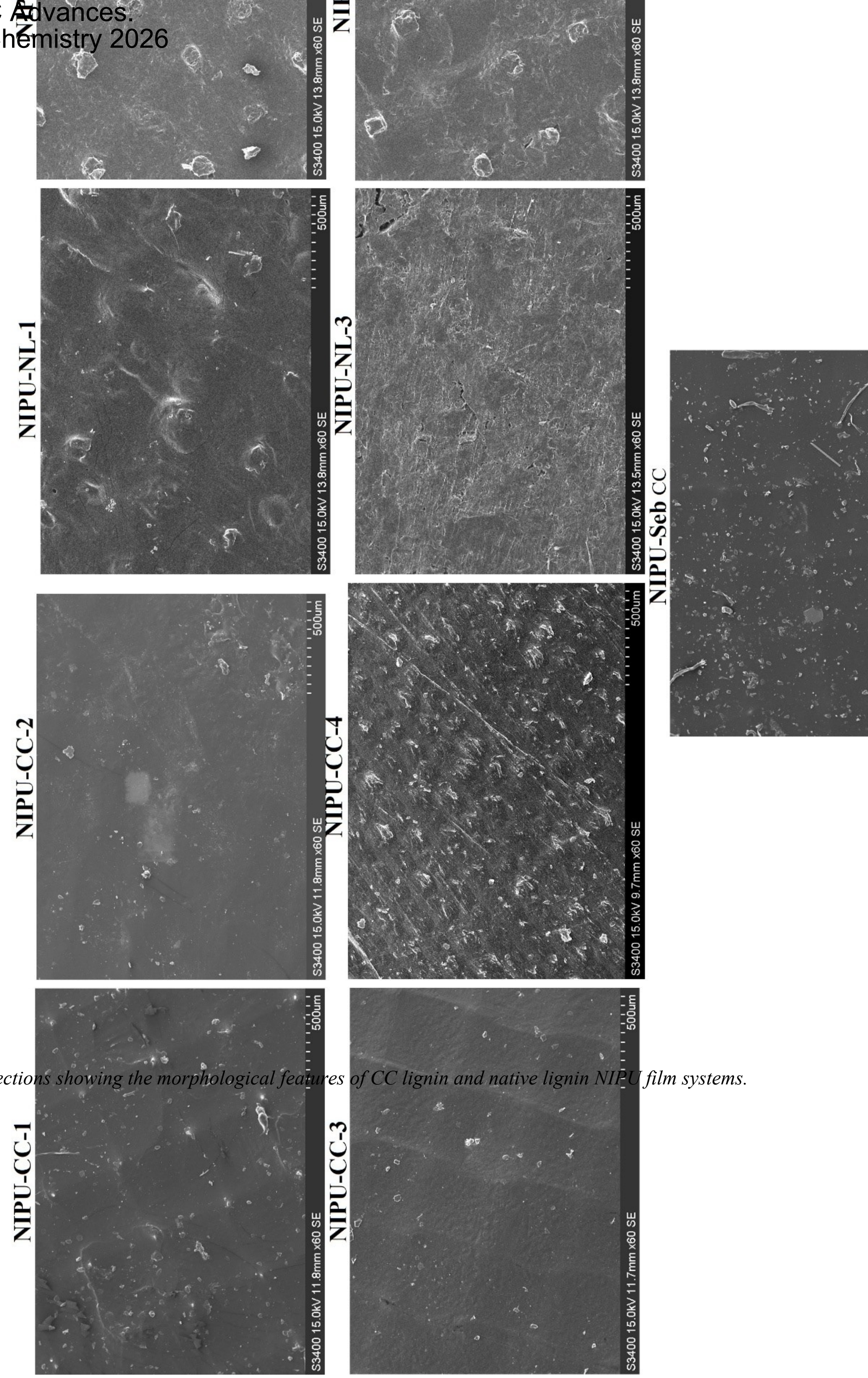

Figure 1: SEM micrographs of NIPU film cross-sections showing the morphological features of CC lignin and native lignin NIPU film systems.
